# Comparison of Contamination Passing Through Iranian and Non- Iranian Filters of Ultra Filtration Dialysis Machines in Patients With Hepatitis C

**DOI:** 10.5812/hepatmon.5912

**Published:** 2013-01-20

**Authors:** Homayoon Bashiri, Hamidreza Omrani, Masood Mami, Mansour Rezaee

**Affiliations:** 1 Department of Internal Medicine, Imam Reza Hospital, Kermanshah University of Medical Sciences, Kermanshah, IR Iran

**Keywords:** Hepatitis C, Ultra-Filtered Liquid, PCR, Dialysis Machine

## Abstract

**Background:**

Hepatitis C is one of the common infectious diseases throughout the world. About 170 million people worldwide are infected with Hepatitis C virus. The most common route of transmission is direct blood-to-blood contacts.

**Objectives:**

This study conducted to compare the amount of contamination might be found in ultra-filtered liquid passed through 2 kinds of filters ps10 (Mediatex, Iran) and Lups (Bio brand, Germany).

**Patients and Methods:**

To achieve the goal, infected dialysis patients in which hepatitis C virus infection was detected by Elisa and PCR were selected.

**Results:**

As shown in data the first stage of PCR test using ps10 filters all samples were negative. In the second step performed in later dialysis steps (with Lups filters), no infection was recorded, too.

**Conclusions:**

Our results showed that dialysis machines do not have an important role in transmission of hepatitis C infection and sanitation control in the environment of dialysis should be emphasized.

## 1. Background

Hepatitis C is one of the common infectious diseases in the world. About 170 million people worldwide are infected with Hepatitis C virus (1). The most common route of transmission is direct blood-to-blood contacts (2). Risk of hepatitis C infection in hemodialysis units is higher than that in normal population (2). This implies that the hemodialysis patients have higher risk in acquisition of hepatitis C. Hepatitis, after cardiovascular diseases and bacterial infections, is the most common complication in hemodialysis patients (2).

In dialysis patients who wait for transplantation, Hepatitis C infection increases risk of graft failure and also the morbidity and mortality during the transplantation (3). Based on the DOPPS prospective study in middle European countries the prevalence of hepatitis C in hemodialysis units is 5-13% (4). Prevalence of hepatitis C in hemodialysis units in Iran is in a range from 5.24% to 50.10% (2). Different risk factors for high prevalence of hepatitis C in dialysis patients have been identified, including transfusion history, duration of dialysis, and adjustments of instrument (5). Despite precise

control of blood products from the presence of hepatitis C and decreased blood transfusions by dialysis patients (due to increased use of erythropoietin), Hepatitis C transmission in dialysis units are still frequently observed (6). Factors caused high prevalence of hepatitis C infection in hemodialysis units are described such as transmission through staffs of dialysis unit and application of common cleansing gauzes on residual blood and hemodialysis patient spread on the bed next to dialysis machine (6). The dialysis machine and its role in transmission has become a major challenge.Prevalence of hepatitis C is higher in dialysis units with common used machines for all referred patients (7). In units with a specific device for dialysis patients infected with hepatitis C, a significant reduction in prevalence of hepatitis C has been recorded (7). Kermanshah (province of Kermanshah, Iran) has two active kidney dialysis centers where all patients either positive or negative for hepatitis C are managed. This study was conducted to determine performance of filters used in dialysis machines to prevent entry of hepatitis C virus to other parts of these machines. Although all filters used for each patient in dialysis machines are disposable, it is possible that hepatitis C virus passes through filters and locates in other parts of machine and likely increases the possibility of infection transmission to following patients.

I) Listen

II) Read phonetically

## 2. Objectives

This study compared the amount of contamination might be found in ultra-filtered liquid passed through 2 kinds of filters ps10 (Mediatex, Iran) and Lup (Bio brand, Germany).

## 3. Patients and Methods

To achieve the goal, dialysis patients in Imam Reza and Imam Khomeini hospitals in Kermanshah who were infected by hepatitis C virus and detected by Elisa and PCR were studied. Statistical analysis for patients with hepatitis C in dialysis units of Imam Reza and Imam Khomeini hospitals was performed. Ultra-filtered liquid of all dialysis patients with hepatitis C was sampled and were analyzed by PCR for infection with Hepatitis C virus. Assayed samples were considered positive if infected with hepatitis C virus and vice versa. Data were shown as copies/ml. Minimum sample size with 95% confidence interval against 80 and 90 was 40 and 53, respectively.

## 3. Results

As shown in Table 1 in the first step of PCR test using ps10 filters all samples were negative. Also, in the second step performed later in dialysis steps (with Lup filters) no infection was recorded. This means that both types of filters have the same characteristics.

**Table 1:** The Results of the Test Participants Based on Iranian and Non-Iranian Filters

Age, y	Gender	Duration of Dialysis, y	Qualitative PCR Results in First Step Iranian	Qualitative PCR Results in the Second Step Non- Iranian
**56**	Male	4	Negative	Negative
**49**	Male	9	Negative	Negative
**38**	Male	6	Negative	Negative
**35**	Female	3	Negative	Negative
**77**	Male	2	Negative	Negative
**33**	Male	8	Negative	Negative
**50**	Male	9	Negative	Negative
**32**	Male	4	Negative	Negative
**80**	Female	9	Negative	Negative
**60**	Male	4	Negative	Negative
**57**	Female	5	Negative	Negative
**57**	Female	6	Negative	Negative
**46**	Male	9	Negative	Negative
**52**	Male	6	Negative	Negative
**32**	Male	3	Negative	Negative
**38**	Male	7	Negative	Negative
**41**	Female	8	Negative	Negative
**41**	Female	8	Negative	Negative
**46**	Male	7	Negative	Negative
**67**	Male	5	Negative	Negative
**60**	Female	5	Negative	Negative
**34**	Female	3	Negative	Negative
**45**	Female	4	Negative	Negative
**53**	Female	6	Negative	Negative
**67**	Female	7	Negative	Negative
**72**	Female	8	Negative	Negative

## 4. Discussion

Prevalence of hepatitis C in dialysis patients is higher than that in general population (4). This could be caused by different factors including lack of attention to important points of bad health, such as neglect in using disposable gloves, using shared heparin vials, etc (8).

Despite of critical points commenced by health personnel and the attention to sanitation of dialysis units for infection control, role of dialysis units in hepatitis C transmission is somehow a challenging subject. ShamshirSaz et al. (2004) showed that dialysis machine plays an important role in transmitting of infection. In patients who used special dialysis units, the prevalence of hepatitis C was 6.1% compared to shared units that was 7.1% (9). However, in some empirical studies it was shown that sterilization could delete virus particles. In such circumstances, employing units as special or shared, did not have important impact on the rate of transmission (10).

On the other hand, José Luis et al. has showed the presence of hepatitis C in ultra-filtered fluid, but some other studies failed to show the role of dialysis machines in transmitting the infection. For example, Claudio and colleagues showed that the final ultra-filtered fluid was free from hepatitis C virus (3); in another study sanitary problems in dialysis settings had the key role in transmission of infection and dialysis machine were not attributed to this issue (10).

Our results showed that the dialysis machines do not have an important role in transmission of hepatitis C infection and considered sanitation in dialysis environment should be emphasized. Considering the fact that the filters of dialysis machine play a key role in reduction of transmission of hepatitis C infection (9), in this study, filters and their roles were evaluated. Although it was advised that other parts of machine was tested in other works, however it requires full information about those parts that was not possible to gain through this study.
